# Comparison of early patient-reported outcomes between uniportal thoracoscopic segmentectomy and wedge resection for peripheral small-sized non-small-cell lung cancer

**DOI:** 10.1186/s13019-024-02635-9

**Published:** 2024-04-15

**Authors:** Yingzhi Zhao, Wenwu Liu, Xin Gao, Kaixin Zhang, Wei Dai, Xing Wei, Haoqian Zheng, Cheng Lei, Hongfan Yu, Qiuling Shi, Qiang Li, Tianpeng Xie

**Affiliations:** 1 Department of Thoracic Surgery, Sichuan Clinical Research Center for Cancer, Sichuan Cancer Hospital & Institute, Sichuan Cancer Center, Affiliated Cancer Hospital of University of Electronic Science and Technology of China, Chengdu, 610041 Sichuan China; 2 Department of Thoracic Surgery, Sichuan Clinical Research Center for Cancer, Sichuan Cancer Hospital & Graduate School, Chengdu Medical college, Chengdu, 610041 Sichuan China; 3 School of Public Health, Chongqing Medical University, Chongqing, 400016 China; 4 State Key Laboratory of Ultrasound in Medicine and Engineering, College of Biomedical Engineering, Chongqing Medical University, Chongqing, 400016 China

**Keywords:** Non-small cell lung carcinoma, Patient-reported outcomes, Pneumonectomy

## Abstract

**Background:**

Analysis of patient-reported outcomes (PROs) offers valuable insights into distinguishing the effects of closely related medical procedures from the patient’s perspective. In this study we compared symptom burden in patients undergoing uniportal thoracoscopic segmentectomy and wedge resection for peripheral small-sized non-small cell lung cancer (NSCLC).

**Methods:**

This study included patients with peripheral NSCLC from an ongoing longitudinal prospective cohort study (CN-PRO-Lung 3) who underwent segmentectomy or wedge resection with tumor diameter ≤ 2 cm and consolidation tumor ratio (CTR) ≤ 0.5. PROs data were collected using the Perioperative Symptom Assessment for Lung Surgery questionnaire pre-operatively, daily post-surgery up to the fourth hospitalization day, and weekly post-discharge up to the fourth week. Propensity score matching and a generalized estimation equation model were employed to compare symptom severity. In addition, short-term clinical outcomes were compared.

**Results:**

In total, data of 286 patients (82.4%) undergoing segmentectomy and 61 patients (17.6%) undergoing wedge resection were extracted from the cohort. No statistically significant differences were found in the proportion of moderate-to-severe symptoms and mean scores for pain, cough, shortness of breath, disturbed sleep, fatigue, drowsiness, and distress during the 4-day postoperative hospitalization or the 4-week post-discharge period before or after matching (all *p* > 0.05). Compared with segmentectomy, wedge resection showed better short-term clinical outcomes, including shorter operative time (*p* = 0.001), less intraoperative bleeding (*p* = 0.046), and lower total hospital costs (*p* = 0.002).

**Conclusions:**

The study findings indicate that uniportal thoracoscopic segmentectomy and wedge resection exert similar early postoperative symptom burden in patients with peripheral NSCLC (tumor diameter ≤ 2 cm and CTR ≤ 0.5).

**Clinical trial registration:**

Not applicable.

**Supplementary Information:**

The online version contains supplementary material available at 10.1186/s13019-024-02635-9.

## Background

Lung cancer has the highest mortality rate among all malignant tumors worldwide [1], and surgical resection remains the gold standard for early-stage lung cancer. In the late 1990s, the advent of health screening and low-dose spiral computed tomography (CT) scanning led to the detection of an increasing number of non-small cell lung cancers (NSCLC) with diameter ≤ 2 cm [2, 3]. Since then, some researchers argue that sublobar resection can yield survival outcomes comparable to those of lobectomy in the treatment of early-stage NSCLC [4, 5], and many studies have explored the optimal surgical procedure for early-stage lung cancer.

The JCOG0201 trial highlighted that imaging diagnosis specificity for non-invasive adenocarcinomas reached 98.7% for tumors ≤ 2 cm in long diameter with a ≤ 0.25 consolidation tumor ratio (CTR) [6]. Building upon these findings, the JCOG0804 study suggested that both wedge resection and segmentectomy can achieve radical cure [7]. Further research [8–10] has indicated that the pathological diagnosis of early lung cancer with nodules ≤ 2 cm in maximum diameter and CTR ≤ 0.5 typically show low invasiveness. The prognosis with wedge resection is not significantly different from that with lobectomy or segmentectomy. Sublobectomy is also recommended for these nodules according to the National Comprehensive Cancer Network guidelines [11]. Despite this, most current studies on sublobar resection for early lung cancer have primarily concentrated on traditional clinical outcomes, including postoperative complications, overall survival, and disease-free survival [9, 12, 13]. These studies have consistently found that patients with early-stage NSCLC, especially those with predominantly ground-glass components, generally enjoy an excellent prognosis and tend to exhibit similar clinical outcomes. Nevertheless, traditional clinical outcomes fail to adequately consider the burden of postoperative symptoms [14]. In recent years, as an increasing emphasis has been placed on the “patient-centered” medical approach in clinical practice [15], there has been a growing focus on patients’ self-perception. Patient-reported outcomes (PROs) have gained prominence in the evaluation of the differences between procedures [14, 16]. PROs offer invaluable insights into patient’s health status and treatment effects, enabling the assessment of symptom burden and functional impairment resulting from similar procedures [17]. This information can assist clinicians in selecting the optimal surgical plan for clinical treatment.

Consequently, evaluating the differences in efficacy between segmentectomy and wedge resection through the evaluation of self-perception of patients after lung cancer surgery may yield fresh insights for clinical decision-making [18]. To the best of our knowledge, no study has yet compared the symptoms and functional status of patients who underwent segmentectomy and wedge resection. Notably, uniportal thoracoscopic surgery has a lighter symptom burden than multiportal thoracoscopic surgery, and has thus been adopted by an increasing number of centers around the world [19]. Therefore, only patients who underwent uniportal thoracoscopic surgery were included in this study. In accordance with previous research and the institutional context [20, 21], the median postoperative hospital stay was 4 days, and patients experienced a significant symptom burden from four days after surgery to one month after discharge. Consequently, this study focused on evaluating the severity of symptoms during this period.

## Methods

### Patients

Patients from an ongoing longitudinal prospective cohort study (CN-PRO-Lung 3) conducted in China between April 2021 and February 2023 were selected for analysis. This study was approved by the Ethics Committee of Sichuan Cancer Hospital (No. SCCHEC-02-2018-043) [22], and all patients provided informed consent to participate.

Based on the JCOG0804 and CALGB140503 trials, as well as existing sublobar resection studies [7, 8, 23], the following inclusion criteria were established for patients: (1) underwent uniportal thoracoscopic anatomical segmentectomy or wedge resection; (2) a pathological diagnosis of NSCLC was made; (3) the tumor was located in the outer third of the lung field on chest CT; and (4) tumors ≤ 2 cm in maximum diameter and CTR ≤ 0.5. Exclusion criteria included: (1) a history of thoracic surgery; (2) a history of neoadjuvant therapy; (3) intraoperative use of both wedge resection and segmentectomy simultaneously; (4) a history of other malignant tumors; and (5) preoperative suspected lymph node metastasis or distant metastasis. To ensure the accuracy of the data, all clinical data and PRO information for the patients were entered and double-checked by two different healthcare providers specializing in thoracic surgery, both of whom received training in uniform criteria before data entry.

### Surgical procedures and postoperative care

Preoperative thin-section lung CT images served as references for both groups, with three-dimensional reconstructions being performed for a small number of patients. All surgical procedures were conducted under general anesthesia, with patients positioned in the lateral decubitus posture, receiving one-lung ventilation on the unaffected side. A surgical incision, approximately 4 cm in length, was made between the fourth or fifth intercostal space, along the anterior and midaxillary lines. The surgery involved the routine use of endoscopic staplers, an ultrasonic scalpel, a coagulation hook, a 30° thoracoscope, and a wound protector. Lesion resection was performed using a thoracoscopic stapler. In our center, wedge resection is performed based on the surgeon’s discretion if the extent of resection can reach 2 cm or larger than the tumor diameter and the tumor location is superficially palpable. Otherwise, segmentectomy is performed to ensure a safe surgical margin. As previously reported, segmentectomy included dissection and division of at least 1 major vascular structure (the segmental artery or vein), as well as the segmental bronchus [24]. After lesion excision, the pulmonary and mediastinal lymph nodes were dissected or sampled in most patients; however, in a small subset of patients with pure ground-glass nodules, only segmentectomy or wedge resection was performed without lymphadenectomy. Upon completing the procedure, a silicone chest tube (20–30 F) was inserted at the original incision site. In one patient, both a silicone chest tube and pigtail chest tube were placed. Intercostal nerve blocks were not administered to any of the patients. A small number of patients were administered hypnotics as needed before surgery, and almost none were administered hypnotics after surgery. Postoperatively, as needed, the patients were routinely administered analgesic pump for pain relief, as well as atomization for relieving cough and asthma; furthermore, they received standardized care such as expectoration care and thromboprophylaxis. ECG monitoring, oxygen saturation monitoring, and the use of urinary catheter were discontinued on the first postoperative day. Patients were appropriately ambulated after urinary catheter removal. Chest tubes were removed when no air leak, hemothorax, or chylothorax was observed, bedside radiographs showed satisfactory residual lung expansion, and the drainage volume was less than 200 mL/d. All patients were discharged after removal of chest tube.

### Outcomes and measures

The severity of each patients’ symptom burden was assessed using the Chinese version of the Perioperative Symptom Assessment for Lung Surgery (PSA-Lung) [25, 26]. This scale was developed and validated in accordance with the recommended standard steps for the development of PRO scales by FDA [27] and was designed to assess the perioperative symptoms related to lung surgery. The scale has been validated to have good reliability and validity [25]. The PSA-Lung comprises seven symptom items (pain, cough, shortness of breath, disturbed sleep, fatigue, drowsiness, and distress) and two functional items (walking difficulty and activity limitation), scored on a scale of 0 to 10. Higher scores indicate a greater symptom burden. This scale reflects the patient’s condition over the past 24 h, featuring straightforward and easily understood content, with a minimal burden during completion, and suitability for frequent measurement of perioperative symptoms in patients undergoing lung surgery.

The proportion of patients with clinically meaningful moderate-to-severe symptoms was compared to evaluate the difference in symptom burden between the two groups. As per previous similar studies, symptoms rated as “moderate to severe” were defined as symptoms with scores ≥ 4 points [21, 28, 29]. Scores recorded on the original scale (0–10 points) were transformed into dichotomous variables (mild, moderate to severe). Furthermore, the mean score of the symptom scale (0–10 points) between the two groups was also compared. PROs data were collected using paper or electronic questionnaires completed by patients. The corresponding time points in these questionnaires were selected for analysis, including pre-surgery, daily assessments up to 4 days post-surgery, and weekly assessments up to 4 weeks after discharge.

The secondary objective aimed to compare short-term postoperative clinical outcomes, encompassing operative time, intraoperative bleeding, chest tube drainage time, length of postoperative hospital stay, incidence of postoperative complications, and total hospital cost. Postoperative complications were assessed using the Clavien–Dindo classification [30], with complications recorded from the time of surgery until discharge. This study reported grade I or higher complications. Demographic and clinical characteristics were collected, including age, sex, educational level, body mass index (BMI), smoking status, American Society of Anesthesiologists Physical Status (ASA-PS) classification [31], comorbidity, percent predicted forced expiratory volume in 1 s (FEV1%), and percent predicted diffusing capacity for carbon monoxide (DLCO%). Surgical conditions included nodule diameter, lymphadenectomy, nodule location, number of chest tubes, nodule histology, and pathological stage of the nodule. Lung cancer staging adhered to the eighth edition of the tumor-node-metastasis (Node) staging [32].

### Statistical analysis

Data analysis was conducted using SPSS 26.0 statistical software. Symptom scores, both pre-surgery and daily up to the fourth day of hospitalization, and then weekly up to the fourth week after discharge, were analyzed for both groups. Descriptive statistics were employed to summarize demographic and clinical characteristics, surgical conditions, and baseline data of the preoperative PROs for both groups. Categorical variables were compared using either the chi-squared test or the two-tailed Fisher’s exact test. Continuous variables were tested for normality using the Kolmogorov–Smirnov test. Continuous variables that followed a normal distribution were assessed using Student ‘s *t*-test, while those that did not were analyzed using the Mann–Whitney U test. To mitigate selection bias between the two groups, propensity score matching was performed at a 1:1 ratio using SPSS 26.0. Each patient’s propensity score was calculated using a logistic regression model adjusted for covariates, including age, sex, educational level, BMI, smoking status, ASA-PS classification, comorbidity, FEV1%, DLCOSB%, nodule diameter, lymphadenectomy, nodule location, number of chest tubes, nodule histology, and nodule pathological stage. The caliper was set to 0.02. To compare the proportion of patients who experienced a moderate-to-severe symptom burden over time between the two surgical groups, a generalized estimating equation model with a logistic link function and a binomial distribution was employed. Furthermore, the mean scores of patients’ symptom burden was also compared between the two groups using a generalized estimating equation model.

All tests were two-sided, and a significance level of *p* < 0.05 was considered statistically significant.

## Results

### Patients’ demographics and clinicopathological characteristics

**Fig. 1:**
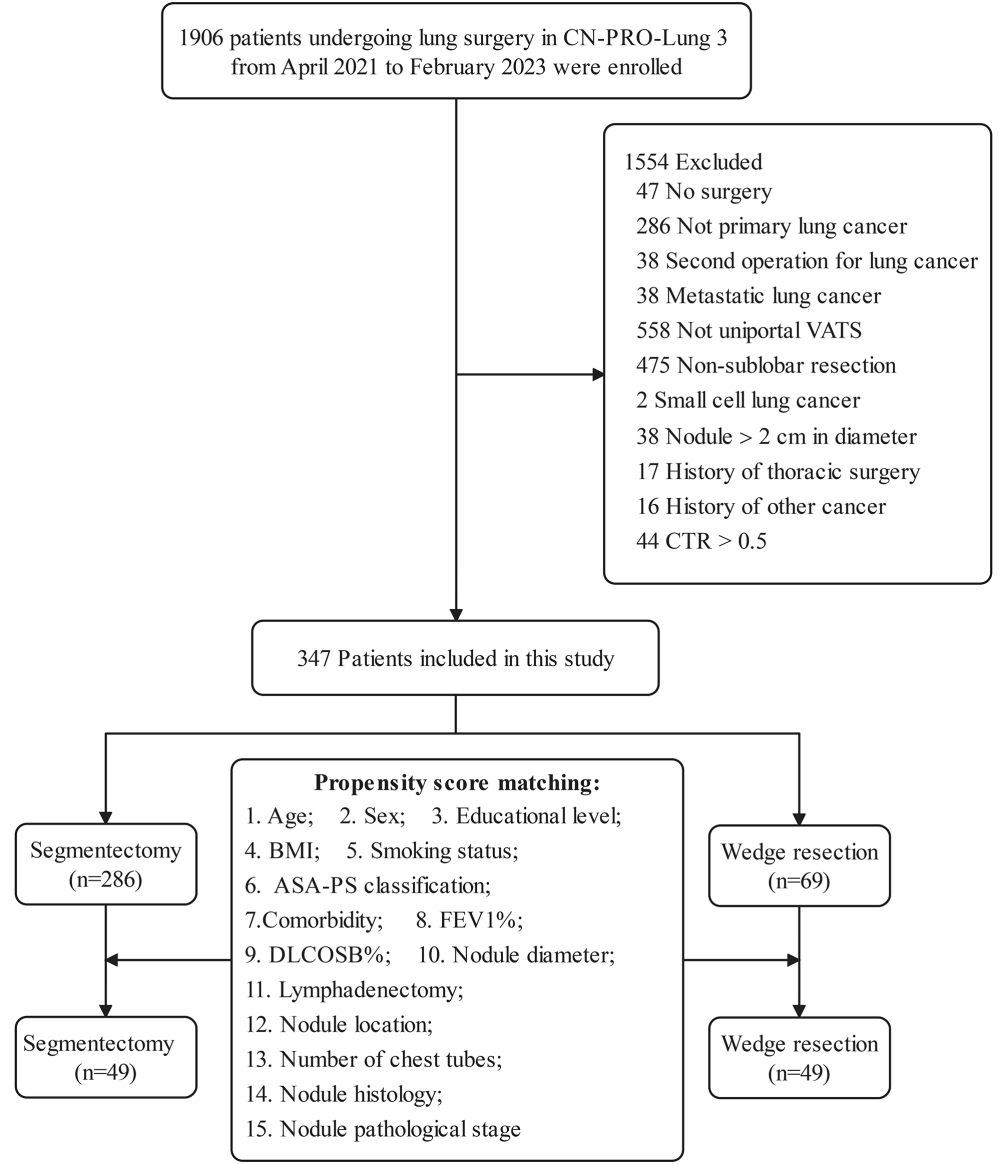
Flow diagram showing patient selection

Figure 1 presents a flowchart illustrating the patient selection process. Among the 1,901 patients who participated in the longitudinal CN-PRO-Lung 3 study conducted from April 2021 to February 2023, 347 met the inclusion and exclusion criteria and were included in the analysis. Among them, 286 underwent segmentectomy (82.4%), while 61 opted for wedge resection (17.6%). The median age of patients in both groups was 49.5 years, reflecting a relatively young population. The majority of patients were female (73.8%) and nonsmokers (84.9%), as detailed in Table 1.

**Table 1:** Demographic and clinical characteristics of the participants

	Before matching				After matching			
	All patients (*n* = 349)	Wedge resection (*n* = 61)	Segmentectomy (*n* = 286)	*p* value	All patients (*n* = 98)	Wedge resection (*n* = 49)	Segmentectomy (*n* = 49)	*p* value
Age (years), median (IQR)	49.50 (39.00–58.00)	50.50(38.50-58.25)	49.00(39.00–58.00)	0.723	51.00 (39.00–58.00)	50.00 (39.00–58.00)	52.00 (39.50–58.50)	0.619
Sex, n (%)				0.999				0.258
Male	91 (26.2)	16 (26.2)	75(26.2)		27 (27.6)	11 (22.4)	16 (32.7)	
Female	256 (73.8)	45 (73.8)	211(73.8)		71 (72.4)	38 (77.6)	33 (67.3)	
Educational level, n (%)				0.776				0.311
≤High school	165 (47.6)	28 (45.9)	137 (47.9)		45 (45.9)	20 (40.8)	25 (51.0)	
>High school	182 (52.4)	33 (54.1)	149 (52.1)		53 (54.1)	29 (59.2)	24 (49.0)	
BMI (kg/m2),median (IQR)	21.88 (20.36–24.23)	21.27 (20.01–23.17)	22.03 (20.44–24.33)	0.136	21.35 (20.02–23.05)	21.23 (19.99–22.75)	21.72 (20.01–23.24)	0.464
Smoking status, n (%)				0.699				0.524
Never	293 (84.9)	54 (88.5)	239 (84.2)		87 (88.8)	45 (91.8)	42 (85.7)	
Current	39 (11.3)	6 (9.8)	33 (11.6)		10 (10.2)	4 (8.2)	6 (12.2)	
Former	13 (3.8)	1 (1.6)	12 (4.2)		1 (1.0)	0 (0.0)	1 (2.0)	
ASA-PS classification, n (%)				1.000				1.000
1	327 (94.8)	58 (95.1)	269 (94.7)		93 (94.9)	46 (93.9)	47 (95.9)	
>1	18 (5.2)	3 (4.9)	15 (5.3)		5 (5.1)	3 (6.1)	2 (4.1)	
Comorbidity, n (%)				1.000				0.610
No	324 (93.9)	57 (93.4)	267 (94.0)		94 (95.9)	46 (93.9)	48 (98.0)	
Yes	21 (6.1)	4 (6.6)	17 (6.0)		4 (4.1)	3 (6.1)	1 (2.0)	
FEV1%, median (IQR), %	94.80 (85.50-104.80)	91.05 (85.35-102.25)	95.15 (85.50-105.18)	0.407	94.40 (86.75-105.76)	92.50 (87.55-102.45)	95.80 (86.05–109.10)	0.893
DLCOSB%, median (IQR), %	92.70 (84.43–104.00)	91.30 (77.85-101.95)	93.45 (84.80-104.45)	0.342	92.35 (84.13-100.85)	91.40 (82.45–100.50)	93.90 (86.45–101.20)	0.412

*IQR* interquartile range; *ASA-PS* American Society of Anesthesiologists physical status, *BMI* body mass index, *DLCO%* percentage of predicted diffusion capacity of carbon monoxide, *FEV1%* percentage of predicted forced expiratory volume in 1 s

Before conducting propensity score matching, there were no discernible differences between the groups in terms of age, sex, educational level, BMI, smoking status, ASA-PS classification, comorbidity, FEV1%, or DLCO%. In terms of surgical procedures, lymph node dissection was performed in the vast majority of patients with segmentectomy (97.2%), whereas the proportion of patients undergoing lymph node dissection during wedge resection decreased significantly (78.7%). Although there was a slight discrepancy in nodule diameter, with the wedge resection group having slightly smaller pulmonary nodules (median nodule diameter, 0.8 cm) than did the segmentectomy group (median nodule diameter, 0.9 cm), this difference did not reach statistical significance. Pathological staging indicated that the majority of patients in both groups were in the early stages of lung cancer, predominantly categorized as stages IA1 and IA2. The detailed intraoperative conditions are presented in Table 2.

**Table 2:** Nodule and operative characteristics before and after matching

	Before matching				After matching			
	All (*n* = 347)	Wedge resection (*n* = 61)	Segmentectomy (*n* = 286)	*p* value	All (*n* = 98)	Wedge resection (*n* = 49)	Segmentectomy (*n* = 49)	*p* value
Nodule diameter (cm), median (IQR)	0.90 (0.70–1.10)	0.80 (0.60–1.10)	0.90 (0.70–1.10)	0.074	0.80 (0.60–1.10)	0.80 (0.60–1.10)	0.80 (0.60–1.10)	0.836
Lymphadenectomy, n (%)				< 0.001				1
Yes	326 (93.9)	48 (78.7)	278 (97.2)		92 (93.9)	46 (93.9)	46 (93.9)	
No	21 (6.1)	13 (21.3)	8 (2.8)		6 (6.1)	3 (6.1)	3 (6.1)	
Nodule location, n (%)				0.529				0.840
Right lung	189 (54.5)	31 (50.8)	158 (55.2)		49 (50.0)	24 (49.0)	25 (51.0)	
Left lung	158 (45.5)	30 (49.2)	128 (44.8)		49 (50.0)	25 (51.0)	24 (49.0)	
Number of chest tubes, n (%)				1				NA
One tube	346 (99.7)	61 (100.0)	285 (99.7)		98 (100.0)	49 (100.0)	49 (100.0)	
Two tubes	1 (0.3)	0 (0.0)	1 (0.3)		NA	NA	NA	
Nodule histology, n (%)				1				NA
Adenocarcinoma	346 (99.7)	61 (100.0)	285 (99.7)		98 (100.0)	49 (100.0)	49 (100.0)	
Non-adenocarcinoma	1 (0.3)	0 (0.0)	1 (0.3)		NA	NA	NA	
Nodule pathological stage, n (%)				0.713				0.694
0	8 (2.3)	1 (1.6)	7 (2.4)		3 (3.1)	1 (2.0)	2 (4.1)	
I A1	253 (72.9)	44 (72.1)	209 (73.1)		72 (73.5)	36 (73.5)	36 (73.5)	
I A2	86 (24.8)	16 (26.2)	70 (24.5)		23 (23.5)	12 (24.5)	11 (22.4)	

*IQR* interquartile range;

Following propensity score-matching, each group consisted of 49 patients. Owing to the low number of cases with two chest tubes and non-adenocarcinoma pathological types, neither group had any cases in these categories after propensity score assignment. Post-matching, the proportion of patients who underwent lymph node dissection was well-balanced between the two groups, with this procedure being performed in 46 patients (93.9%) across both groups. No statistically significant differences were identified in the demographic and clinical characteristics or intraoperative conditions between the two groups after matching.

### Patient-reported symptoms

Before conducting propensity score matching, the majority of the 347 patients had completed preoperative baseline PSA-lung data, with a completion rate of 98.6%. However, the completion rates for PSA-Lung at 4 days after the operation and 4 weeks after discharge ranged from 63.8 to 90.2% and 63.4–74.1%, respectively (Data presented in a table: Additional File 1). Evaluation of the seven symptoms within PSA-Lung (pain, cough, shortness of breath, disturbed sleep, fatigue, drowsiness, and distress) revealed no differences in the proportion of moderate-to-severe symptoms and mean scores when comparing baseline data (Additional File 2, Additional File 3). No significant differences were observed between the two groups in terms of the proportion of moderate-to-severe scores for the seven symptoms: pain (*p* = 0.887), cough (*p* = 0.254), shortness of breath (*p* = 0.929), disturbed sleep (*p* = 0.372), fatigue (*p* = 0.695), drowsiness (*p* = 0.692), and distress (*p* = 0.201) between days 1 and 4 of postoperative hospitalization and weeks 1 to 4 after discharge. Moreover, the mean scores of the seven symptoms were not significantly different between the two groups (Additional File 4).

**Fig. 2:**
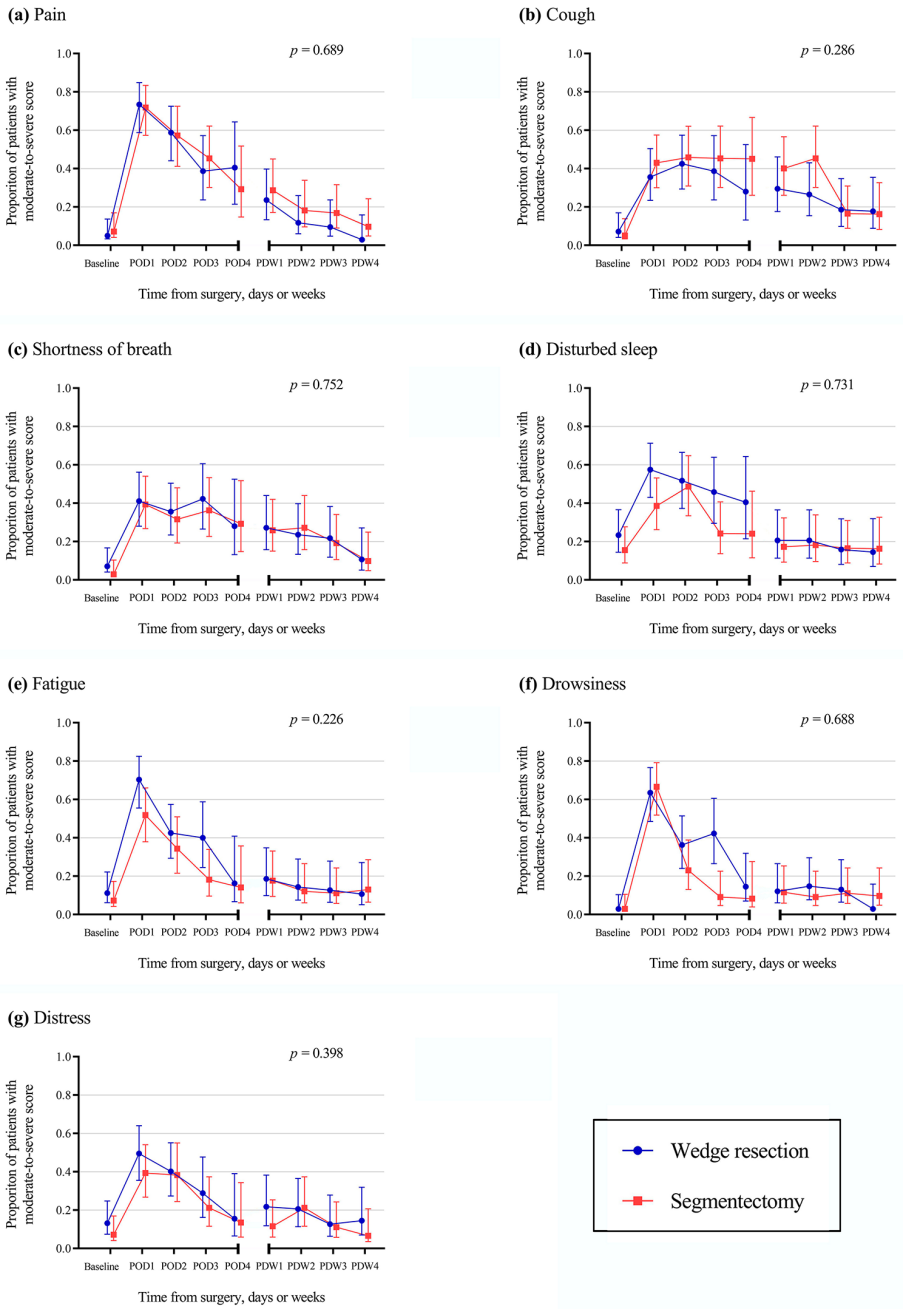
Proportion of patients with moderate to severe symptoms after propensity score matching *POD*, Postoperative day; *PDW*, Post-discharge week

After propensity score matching, the PSA-Lung completion rates for the 98 patients were 99.9%, 68.8–90.8%, and 58.2–70.4% at preoperative baseline, 4 days postoperative hospitalization, and 4 weeks after discharge, respectively, data presented in a table (Additional File 5). Among the seven symptoms analyzed, no differences emerged between the groups with regard to the proportion of moderate-to-severe symptoms and mean scores when comparing baseline data (Additional File 6, Additional File 7). Likewise, no significant between-group differences were observed for the proportion of moderate-to-severe scores related to pain (*p* = 0.689), cough (*p* = 0.286), shortness of breath (*p* = 0.752), disturbed sleep (*p* = 0.731), fatigue (*p* = 0.226), drowsiness (*p* = 0.688), and distress (*p* = 0.398) during the period encompassing 4 days of postoperative hospitalization and 4 weeks after discharge (Fig. 2). Figure 2 depicts the difference in the proportion of moderate-to-severe symptoms between the four time points including disturbed sleep, fatigue, and drowsiness on the third day after surgery, and cough during the second week after discharge. Hence, a Chi-square analysis was performed separately for symptoms at these four time points; the analysis revealed that disturbed sleep (*p* = 0.069) on postoperative day 3, fatigue (*p* = 0.051) on postoperative day 3, and cough during second week after discharge (*p* = 0.100) were not statistically significant. Only drowsiness (*p* < 0.05) was significantly different on the third postoperative day. In addition, no differences were found between the groups with regard to the mean scores of the seven symptoms after matching. Data is presented in a table (Additional File 8).

### Short-term clinical outcomes

Before conducting propensity score matching, wedge resection was associated with better perioperative outcomes than segmentectomy, including a shorter operative time (*p* = 0.001), less intraoperative bleeding (*p* < 0.001), and lower total hospital costs (*p* < 0.001). However, no significant differences were observed in chest tube drainage time (*p* = 0.706), median postoperative hospital stay (*p* = 0.996), or the incidence of postoperative complications (*p* = 0.377) between the two groups (Table 3).

**Table 3:** Traditional clinical outcomes

	Before matching			After matching		
	Wedge resection (*n* = 61)	Segmentectomy (*n* = 286)	*p* value	Wedge resection (*n* = 49)	Segmentectomy (*n* = 49)	*p* value
Operative time (min), median (IQR)	50.00 (35.00–70.00)	70.00 (55.00-105.00)	< 0.001	50.00 (35.00–70.00)	65.00 (55.00-105.00)	0.001
Intraoperative bleeding (ml), median (IQR)	20.00 (20.00–50.00)	50.00 (20.00–50.00)	< 0.001	20.00 (20.00–50.00)	50.00 (20.00–50.00)	0.046
Drainage time (days), median (IQR)	2.00(2.00–3.00)	2.00(2.00–3.00)	0.706	2.00(2.00–3.00)	2.00(2.00–3.00)	0.454
Postoperative hospital stay (days), median (IQR)	4.00(3.00–4.00)	4.00 (3.00–4.00)	0.996	4.00(3.00–4.00)	4.00 (3.00–4.00)	0.762
Complications, n (%)			0.377			0.475
No	60 (98.4)	271 (94.8)		49 (100.0)	47 (95.9)	
Yes^a^	1 (1.6)	15 (5.2)		0 (0.0)	2 (4.1)	
Total cost (RMB), median (IQR)	37021.89 (31981.50-41055.42)	404480.16 (35124.37-46639.53)	< 0.001	37021.98 (32468.05-41557.81)	40834.01 (37071.26-47602.72)	0.002

*IQR* interquartile range; ^a^Grade I or higher complications according to the Clavien–Dindo classification are reported

Following propensity score matching analysis, similar results were obtained. Significant differences were observed between the two groups in terms of median operation time (*p* < 0.001), intraoperative bleeding (*p* = 0.046), and total hospital cost (*p =* 0.002). However, no significant differences were found between groups in the chest tube drainage time (*p* = 0.454), median postoperative hospital stay (*p* = 0.762), or incidence of postoperative complications *(p* = 0.475).

## Discussion

To the best of our knowledge, no studies have yet reported on the symptom burden following uniportal thoracoscopic segmentectomy and wedge resection for early NSCLC. In this study, the symptom burden of patients who underwent segmentectomy and wedge resection showed similar results based on PROs during the 1–4 days of postoperative hospitalization or 1–4 weeks after discharge. No significant differences were found between the two groups even after propensity score matching. In traditional clinical outcomes, patients who underwent wedge resection showed better perioperative outcomes, including shorter operative time, reduced intraoperative bleeding, and lower total hospital costs, both before and after matching.

In a previous study, we presented the PROs for thoracoscopic lobectomy and segmentectomy in early lung cancer cases, [21] where both procedures exhibited similar early symptom burden and functional impairments. This study extends our previous research by delving into the disparity between segmentectomy and wedge resection using PRO data in patients with early-stage lung cancer, addressing an existing research gap. By comparing the pros and cons of these two procedures in terms of PROs, we contribute further evidence to support clinical decision-making. Importantly, no significant differences in PROs were identified between the two procedures in this study.

Postoperative pain in patients primarily stemmed from the chest wall incision [33]. All patients in this study underwent uniportal thoracoscopy with a single incision, with relatively standardized dimensions (approximately 4 cm) and location (between the fourth and fifth intercostal spaces, along the anterior axillary line to the midaxillary line), suggesting that postoperative pain resulting from segmentectomy and wedge resection may be comparable. Regarding cough, the proportion of patients with moderate-to-severe cough was higher after segmentectomy than after wedge resection at most time points, as shown by the line graphs. This discrepancy may be attributed to the more frequent manipulation of lung tissue during segmentectomy, along with the duration of endotracheal intubation during surgery. While cough symptoms were more pronounced in patients undergoing segmentectomy, the difference did not reach statistical significance. An extended follow-up period may be necessary to elucidate potential variations between the two groups. In terms of shortness of breath, neither group showed a large difference, suggesting that both segmentectomy and sublobar resections result in minimal damage to lung parenchyma. Furthermore, patients in both groups were younger and displayed robust pulmonary function compensation, which may explain why shortness of breath may not differ significantly between the groups. Previous studies have indicated that pain, fatigue, disturbed sleep, and distress form a symptom cluster [34] following lung cancer surgery, with these symptoms often interacting with one another [35]. Sleep quality, for example, can be significantly impacted by postoperative pain [36]. When sleep quality decreases, patients experience increased fatigue and drowsiness, subsequently affecting their mental well-being and leading to distress [37]. It is possible that the absence of significant differences in pain between the two groups contributed to the similarity in the effects of pain-related symptoms between them. Additionally, distress is employed to describe the psychological status of patients with lung cancer [38]. Patients were well-informed during preoperative discussions that the vast majority of peripheral lung cancers ≤ 2 cm in diameter and ≤ 0.5 in CTR were early-stage and clinically curable, with essentially no need for subsequent treatment. Thus, the psychological burden of patients was relatively light and similar between the two groups. Overall, it is plausible that the minimally invasive uniportal thoracoscopic technique itself substantially reduces the burden of postoperative symptoms in patients [19]. Consequently, small differences between various sublobectomy procedures may not be statistically discernible.

Notably, drowsiness on the third postoperative day was statistically significantly different between the two groups. It indicates that the symptom burden of drowsiness on the third postoperative day in patients undergoing wedge resection is heavier than that in segmentectomy; however, this is difficult to clinically explain. This could have possibly been a chance error owing to the small sample size. Hence, we performed a separate chi-square analysis for each time point of the seven symptoms after matching, with a total of 56 chi-square analyses, which revealed a significant difference only in the drowsiness on the third postoperative day, and we considered one chance error acceptable. Furthermore, there was no statistical difference between the two groups in the overall analysis of the drowsiness longitudinal data. It is necessary to conduct future studies with larger sample size to verify whether the longitudinal data is consistent with the symptom burden at a certain point in time.

Pulmonary segments constitute independent anatomical structures and functional units [39], with each segment possessing its own lymphatic drainage, segmental bronchi, arteries, and veins. A substantial number of patients exhibit vascular and bronchial variations [40, 41], resulting in prolonged operation times when accurately locating the target lung segment, vessels, and bronchi. Segmentectomy requires adequate mobilization of the deep vessels and bronchi, leading to increased damage to surrounding tissues and, consequently, elevated intraoperative bleeding [42]. Notably, the amount of bleeding was small in both procedures, and although segmentectomy had a statistically significantly greater amount of bleeding than wedge resection, the actual clinical significance of this difference may be small. Moreover, segmentectomy presents more technical challenges compared to wedge resection, requiring more stapler reloads and hemostatic materials during surgery. Consequently, the overall hospitalization cost associated with segmentectomy is higher than that associated with wedge resection. Nevertheless, no significant differences were observed between the two groups in terms of chest tube drainage time, duration of postoperative hospital stay, or the incidence of postoperative complications. These findings suggest that wedge resection is as safe as segmentectomy in terms of short-term prognosis, and may constitute a secure and feasible surgical approach for early lung cancer with low metastasis risk. This finding aligns with that previously reported [9, 12, 13].

Compared to previous reports, our study has several strengths. First, our research was patient-centered, relying on PROs for more sensitive data that can uncover direct differences between the two surgical modalities [17, 19]. This approach compensates for traditional data limitations. Second, we transformed the 0–10 scores recorded on the form into dichotomous variables (mild and moderate to severe), and compared different proportions of patients with moderate-to-severe symptoms between groups, which may achieve clinical significance [19]. Third, we employed PSA-Lung, a concise and relatively unburdensome tool for patients, for longitudinal measurements at multiple timepoints post-surgery, thereby reflecting patient status. In previous studies, assessments of time points were limited and the intervals were long, leading to the inadvertent omission of postoperative PRO data [43, 44].

Nevertheless, our study has some limitations that should be mentioned. Firstly, this was an observational study; hence, several confounding factors were introduced. Despite employing propensity score matching based on baseline data, potential patient-related biases may not have been entirely eliminated. Further validation through RCTs is required in the future. Second, the patients in our study were predominantly young women and non-smokers, which represents the characteristic lung cancer population in our region [45] and may not be a true representation of the European and American populations [1, 46]. To enhance the generalizability of our results, future studies should validate our findings in different regions, populations, and multi-institutional settings. Third, the relatively small sample size in our study may not fully capture symptom burden and trends among the target group, potentially rendering the observed differences or associations statistically insignificant. As such, increasing the cohort size in future research could improve sample representativeness and result stability. Fourth, we did not statistically analyze the postoperative care and medication of wedge resection versus segmentectomy to investigate the impact on symptoms, although we administered standardized postoperative care and conventional medication. It is necessary to take these factors into account in future study designs.

## Conclusions

The results of this study revealed that uniportal thoracoscopic segmentectomy and wedge resection exhibited comparable early postoperative symptom burden for patients with peripheral NSCLC nodules ≤ 2 cm in diameter and CTR ≤ 0.5, while wedge resection demonstrated superior short-term clinical outcomes in comparison to segmentectomy. These study outcomes support the utilization of wedge resection in this specific patient cohort.

### Electronic supplementary material

Below is the link to the electronic supplementary material.

Supplementary Material 1

## Data Availability

De-identified participant data are available on reasonable request to the corresponding author starting from 6 months after this study is published.

## Abbreviations

PROs: patient-reported outcomes
NSCLC: non-small cell lung cancer
CT: computed tomography
CTR: consolidation tumor ratio
NCCN: National Comprehensive Cancer Network
ASA-PS: American Society of Anesthesiologists Physical Status
FEV1%: percentage of predicted forced expiratory volume in 1 s
DLCO%: percentage of predicted diffusion capacity of carbon monoxide
PSA-Lung: Perioperative Symptom Assessment for Lung Surgery
