# Supplementary material for: Comparison of early patient-reported outcomes between uniportal thoracoscopic segmentectomy and wedge resection for peripheral small-sized non-small-cell lung cancer
Source: J Cardiothorac Surg. 2024 Apr 15;19:215. doi: 10.1186/s13019-024-02635-9 (PMC11017554; doi:10.1186/s13019-024-02635-9)
Supplement: Supplementary file 1 — Supplementary Material 1 [file 13019_2024_2635_MOESM1_ESM.docx]

**Additional Files**

**Additional File1.** Completion rates of PSA-Lung data before matching

**Additional File2.** Preoperative baseline data of PROs before matching

**Additional File3.** Preoperative baseline mean score of PROs before matching

**Additional File4.** Before propensity score matching, generalized estimating equation models were used to compare the difference in mean scores of PROs between the two groups at nine time points

**Additional File5.** Completion rates of PSA-Lung data after matching

**Additional File6.** Preoperative baseline data of PROs after matching

**Additional File7.** Preoperative baseline mean score of PROs after matching

**Additional File8.** After propensity score matching, generalized estimating equation models were used to compare the difference in mean scores of PROs between the two groups at nine time points

| **Additional File1** Completion rates of PSA-Lung data before matching | | | |
| --- | --- | --- | --- |
| Time Points | Available, n | Scheduled, n | Percentage (%) |
| Baseline | 342 | 347 | 0.986 |
| POD 1 | 313 | 347 | 0.902 |
| POD 2 | 286 | 347 | 0.824 |
| POD 3 | 209 | 311 | 0.672 |
| POD 4 | 88 | 138 | 0.638 |
| Discharge week 1 | 257 | 347 | 0.741 |
| Discharge week 2 | 248 | 347 | 0.715 |
| Discharge week 3 | 253 | 347 | 0.729 |
| Discharge week 4 | 220 | 347 | 0.634 |
| *PSA-Lung,* Perioperative Symptom Assessment for Lung Surgery; *POD,* postoperative day. | | | |

| **Additional File2** Preoperative baseline data of PROs before matching | | | | |
| --- | --- | --- | --- | --- |
|  | Wedge resection  n (%) | Segmentectomy  n (%) | All  n (%) | *p* value |
| Pain |  |  |  | 1.000 |
| Mild | 60 (98.4) | 274 (97.5) | 334 (97.7) |  |
| Moderate to severe | 1 (1.6) | 7 (2.5) | 8 (2.3) |  |
| Cough |  |  |  | 0.539 |
| Mild | 57 (95.0) | 272 (97.5) | 329 (97.1) |  |
| Moderate to severe | 3 (5.0) | 7 (2.5) | 10 (2.9) |  |
| Shortness of breath |  |  |  | 0.805 |
| Mild | 59 (96.7) | 275 (98.2) | 334 (97.9) |  |
| Moderate to severe | 2 (3.3) | 5 (1.8) | 7 (2.1) |  |
| Disturbed sleep |  |  |  | 0.814 |
| Mild | 50 (82.0) | 233 (83.2) | 283 (83.0) |  |
| Moderate to severe | 11 (18.0) | 47 (16.8) | 58 (17.0) |  |
| Fatigue |  |  |  | 0.667 |
| Mild | 56 (91.8) | 263 (94.3) | 319 (93.8) |  |
| Moderate to severe | 5 (8.2) | 16 (5.7) | 21 (6.2) |  |
| Drowsiness |  |  |  | 1.000 |
| Mild | 58 (96.7) | 271 (96.8) | 329 (96.8) |  |
| Moderate to severe | 2 (3.3) | 9 (3.2) | 11 (3.2) |  |
| Distress |  |  |  | 0.732 |
| Mild | 55 (90.2) | 258 (92.5) | 313 (92.1) |  |
| Moderate to severe | 6 (9.8) | 21 (7.5) | 27 (7.9) |  |
| *PROs,* Patient-reported outcomes. | | | | |

| **Additional File3** Preoperative baseline mean score of PROs before matching | | | | |
| --- | --- | --- | --- | --- |
|  | Wedge resection | Segmentectomy | All | p value |
| Mean (SD) score at baseline |  |  |  |  |
| Pain | 0.28 (0.839) | 0.30 (0.909) | 0.30 (0.896) | 0.851 |
| Cough | 0.63 (1.301) | 0.60 (1.078) | 0.60 (1.119) | 0.828 |
| Shortness of breath | 0.64 (1.170) | 0.36 (0.886) | 0.41 (0.947) | 0.087 |
| Disturbed sleep | 1.85 (2.190) | 1.49 (1.944) | 1.56 (1.991) | 0.202 |
| Fatigue | 0.80 (1.459) | 0.79 (1.499) | 0.79 (1.490) | 0.944 |
| Drowsiness | 0.60 (1.224) | 0.69 (1.308) | 0.68 (1.293) | 0.614 |
| Distress | 0.98 (1.936) | 0.90 (1.509) | 0.91 (1.591) | 0.710 |
| *PROs*, Patient-reported outcomes; *SD*, standard deviation. | | | | |

| **Additional File4** Before propensity score matching, generalized estimating equation models were used to compare the difference in mean scores of PROs between the two groups at nine time points | | | | |
| --- | --- | --- | --- | --- |
|  | Wedge resection | Segmentectomy | All | *p* value |
| Mean (SD) score^a^ |  |  |  |  |
| Pain | 2.55 (2.408) | 2.57 (2.341) | 2.57 (2.352) | 0.772 |
| Cough | 2.35 (2.227) | 2.52 (2.104) | 2.49 (2.126) | 0.355 |
| Shortness of breath | 2.30 (2.261) | 2.20 (2.124) | 2.21 (2.148) | 0.656 |
| Disturbed sleep | 2.59 (2.409) | 2.32 (2.250) | 2.37 (2.280) | 0.276 |
| Fatigue | 2.21 (2.198) | 2.18 (2.189) | 2.19 (2.190 )_ | 0.971 |
| Drowsiness | 2.02 (2.297) | 2.00 (2.224) | 2.01 (2.236) | 0.941 |
| Distress | 1.85 (2.095) | 1.83 (2.072) | 1.84 (2.076) | 0.990 |
| *PROs*, Patient-reported outcomes; *SD*, standard deviation  ^a^Mean score refers to mean score of nine time points | | | | |

| **Additional File5** Completion rates of PSA-Lung data after matching | | | |
| --- | --- | --- | --- |
| Time Points | Available, n | Scheduled, n | Percentage (%) |
| Baseline | 97 | 98 | 0.990 |
| POD 1 | 89 | 98 | 0.908 |
| POD 2 | 78 | 98 | 0.796 |
| POD 3 | 61 | 85 | 0.718 |
| POD 4 | 33 | 48 | 0.688 |
| Discharge week 1 | 69 | 98 | 0.704 |
| Discharge week 2 | 68 | 98 | 0.694 |
| Discharge week 3 | 69 | 98 | 0.704 |
| Discharge week 4 | 57 | 98 | 0.582 |
| *PSA-Lung,* Perioperative Symptom Assessment for Lung Surgery; *POD,* postoperative day. | | | |

| **Additional File6** Preoperative baseline data of PROs after matching | | | | |
| --- | --- | --- | --- | --- |
|  | Wedge resection  n (%) | Segmentectomy  n (%) | All  n (%) | *p* value |
| Pain |  |  |  | 0.986 |
| Mild | 48 (98.0) | 46 (95.8) | 94 (96.9) |  |
| Moderate to severe | 1 (2.0) | 2 (4.2) | 3 (3.1) |  |
| Cough |  |  |  | 1 |
| Mild | 46 (95.8) | 47 (97.9) | 93 (96.9) |  |
| Moderate to severe | 2 (4.2) | 1 (2.1) | 3 (3.1) |  |
| Shortness of breath |  |  |  | 0.495 |
| Mild | 47 (95.9) | 48 (100.0) | 95 (97.9) |  |
| Moderate to severe | 2 (4.1) | 0 (0.0) | 2 (2.1) |  |
| Disturbed sleep |  |  |  | 0.294 |
| Mild | 39 (79.6) | 42 (87.5) | 81 (83.5) |  |
| Moderate to severe | 10 (20.4) | 6 (12.5) | 16 (16.5) |  |
| Fatigue |  |  |  | 0.712 |
| Mild | 45 (91.8) | 45 (95.7) | 90 (93.8) |  |
| Moderate to severe | 4 (8.2) | 2 (4.3) | 6 (6.3) |  |
| Drowsiness |  |  |  | NA |
| Mild | 48 (100.0) | 47 (100.0) | 95 (100.0) |  |
| Moderate to severe | NA | NA | NA |  |
| Distress |  |  |  | 0.449 |
| Mild | 44 (89.8) | 46 (95.8) | 90 (92.8) |  |
| Moderate to severe | 5 (10.2) | 2 (4.2) | 7 (7.2) |  |
| *PROs,* Patient-reported outcomes. | | | | |

| **Additional File7**  Preoperative baseline mean score of PROs after matching | | | | |
| --- | --- | --- | --- | --- |
|  | Wedge resection | Segmentectomy | All | *p* value |
| Mean (SD) score at baseline |  |  |  |  |
| Pain | 0.29 (0.890) | 0.33 (0.930) | 0.31 (0.906) | 0.797 |
| Cough | 0.60 (1.317) | 0.50 (1.010) | 0.57 (1.167) | 0.795 |
| Shortness of breath | 0.59 (1.206) | 0.29 (0.617) | 0.44 (0.968) | 0.126 |
| Disturbed sleep | 2.08 (2.299) | 1.29 (1.868) | 1.69 (2.123) | 0.067 |
| Fatigue | 0.71 (1.399) | 0.60 (1.173) | 0.66 (1.288) | 0.655 |
| Drowsiness | 0.48 (1.010) | 0.74 (1.093) | 0.61 (1.055) | 0.222 |
| Distress | 1.08 (1.988) | 0.77 (1.171) | 0.93 (1.635) | 0.350 |
| *PROs*, Patient-reported outcomes; *SD*, standard deviation | | | | |

| **Additional File8** After propensity score matching, generalized estimating equation models were used to compare the difference in mean scores of PROs between the two groups at nine time points | | | | |
| --- | --- | --- | --- | --- |
|  | Wedge resection | Segmentectomy | All | *p* value |
| Mean (SD) score^a^ |  |  |  |  |
| Pain | 2.51 (2.362) | 2.61 (2.410) | 2.56 (2.385) | 0.568 |
| Cough | 2.40 (2.271) | 2.44 (1.928) | 2.42 (2.102) | 0.869 |
| Shortness of breath | 2.25 (2.239) | 2.12 (1.981) | 2.18 (2.111) | 0.645 |
| Disturbed sleep | 2.69 (2.433) | 2.12 (2.106) | 2.40 (2.288) | 0.081 |
| Fatigue | 2.24 (2.228) | 1.87 (1.974) | 2.05 (2.109) | 0.136 |
| Drowsiness | 2.06 (2.331) | 1.81 (2.044) | 1.93 (2.191) | 0.336 |
| Distress | 2.00 (2.133) | 1.73 (1.980) | 1.86 (2.060) | 0.538 |
| *PROs*, Patient-reported outcomes; *SD*, standard deviation  ^a^Mean score refers to mean score of nine time points | | | | |
